# Evaluation of Lipid Profile in Patients with Cherry Angioma: A Case-Control Study in Guilan, Iran

**DOI:** 10.1155/2018/4639248

**Published:** 2018-05-13

**Authors:** Abbas Darjani, Rana Rafiei, Sareh Shafaei, Elahe Rafiei, Hojat Eftekhari, Narges Alizade, Kaveh Gharaei nejad, Behnam Rafiee, Sara Najirad

**Affiliations:** ^1^Skin Research Center, Dermatology Department, Guilan University of Medical Sciences, Razi Hospital, Sardare Jangal Street, Rasht, Iran; ^2^Fellowship of Dermatopathology, Skin Research Center, Dermatology Department, Guilan University of Medical Sciences, Razi Hospital, Sardare Jangal Street, Rasht, Iran; ^3^Razi Clinical Research Development Center, Guilan University of Medical Sciences, Rasht, Iran; ^4^Department of Pathology, NYU Winthrop Hospital, 222 Station Plaza, No. 620, Mineola, NY 11501, USA; ^5^Department of Internal Medicine, Nassau University Medical Center, 2201 Hempstead Turnpike, East Meadow, NY 11554, USA

## Abstract

**Background:**

Cherry angioma is the most common type of acquired cutaneous vascular proliferation which would increase with aging due to some angiogenic factors but the exact pathogenesis is unknown. Usually angiogenic factors are synthesized in human body to compensate occlusive effects of atherogenic agents such as serum lipids. Our hypothesis was that increased levels of these angiogenic factors could be a trigger for development of cherry angioma. This study has been designed to compare frequency of dyslipidemia in subjects with and without cutaneous cherry angioma.

**Methods:**

In this case-control study, 122 cases with cherry angioma and 122 control subjects without cherry angioma were enrolled. Demographic characteristics, number of the cherry angioma lesions, and serum lipid profile were collected for all subjects. The data was analyzed using SPSS 18 software.

**Results:**

Mean levels of the total cholesterol, triglyceride, low-density lipoprotein, and high-density lipoprotein were higher in patients with cherry angioma compared to control subjects in which differences were significant for total cholesterol, low-density lipoprotein, and triglyceride (*P* < 0.05) but not for high-density lipoprotein level.

**Conclusion:**

Serum lipids may have a role in producing angiogenic factors and development of cherry angioma and it seems logical to evaluate lipid profile in these cases.

## 1. Introduction

Cherry angioma (CA) or senile angioma is the most common type of acquired benign vascular proliferation which usually presents as nonblanching red papules on the acral and truncal areas [1]. CA has been seen in 2% of children, 50% of adults, and 50%–75% of people aged older than 75 years [1–4]. It has a polygenic mode of inheritance. These lesions often have no symptoms and patients are always concerned about the increasing number of the lesions, risk of malignancy, and their cosmetic aspects [1]. CA develops due to abnormal proliferation of well differentiated endothelial cells [5]. The exact pathogenesis is unknown. Hyperprolactinemia, pregnancy, human herpesvirus 8 (HHV-8) infection, mustard gas poisoning, immunosuppression induced by cyclosporine, underlying malignancy, some chemokines, and bromide and 2-butoxyethanol exposure all have been incriminated [6–11]. These agents would be associated with eruptive form of CA but in noneruptive form of CA; the lesions gradually increase with aging and they are more and larger in diabetic patients [1, 12]. Our hypothesis was that some angiogenic factors are synthesized in the body to compensate occlusive effects of atherogenic agents. These angiogenic factors could be a trigger for development of CA. Serum lipids are one of the most important atherogenic agents [11, 13].

This research was designed to determine possible association between CA and dyslipidemia. If we could find any association, CA might be considered as a cutaneous marker of dyslipidemia and these patients should be screened earlier to prevent cardiovascular diseases.

## 2. Materials and Methods

This study was designed as an age-matched case-control study at Skin Research Center of Guilan University of Medical Sciences, Razi Hospital, Rasht, from June 2016 to March 2017.

This study included 244 volunteers, 122 cases with CA and 122 healthy control subjects without CA, randomly selected from our patients who visited the hospital for aesthetic reasons. Final sample size was calculated after a primary pilot study. Both groups were frequency-matched in age groups (30–39; 40–49; 50–59; …, 80–89 years).

All patients aged ≥30 years who did not meet our exclusion criteria and had consent for body examination and doing laboratory evaluation were included.

We excluded some cases as follows to decrease confounding factors that may affect serum lipid levels: patients with drug history of agents which could be able to change lipid profile such as statin group, fibrate group, beta blockers, steroidal hormones, contraceptive pills, cyclosporine, oral retinoids, and diuretics; pregnancy or lactation, smoking, alcoholism, diabetes, history of mustard gas poisoning, hypertension, underlying malignancy, thyroid dysfunction, eruptive form of CA; patients who had very high lipid levels in the form of familial dyslipidemia; and patients with chronic inflammatory skin diseases such as lichen planus or psoriasis.

CA was diagnosed based on the physical examination performed by an individual dermatologist without skin biopsy.

Demographic characteristics of participants (age, sex, and Fitzpatrick skin phototype) and number of the cherry lesions were recorded in the first visit. Lipid profile including triglyceride (TG), cholesterol (CHOL), low-density lipoprotein (LDL), and high-density lipoprotein (HDL) level was collected for both groups. Lipid profile was evaluated in venous blood samples drawn after 12 h fasting in an individual laboratory by Hitachi analyzer 717 made in Japan.

Examination of the patients was conducted according to the declaration of Helsinki principles with informed consent. For patient comfort, scalp and genital area were not examined. This research project was approved by Ethics Committee of Guilan University of Medical Sciences (Code: IR.GUMS.REC.1395.204). The data of our manuscript is available.

Dyslipidemia was defined as TG level ≥ 150 mg/dl (hypertriglyceridemia) or total cholesterol level ≥ 200 mg/dl (hypercholesterolemia) or LDL level ≥ 130 mg/dl or HDL level < 40 mg/dl for men and <50 mg/dl for women, so if a patient had one or more of these abnormalities, they were categorized as having dyslipidemia [14].

Quantitative variables were described using mean and standard deviation and qualitative data were reported by number and percentage. For the comparison of frequencies, the chi-square test or Fisher's exact test was used and, for the comparison of continuous variables, Mann–Whitney and Kruskal–Wallis test were applied in both groups. All statistical tests were performed in SPSS 18 software. All *P* values were two-sided; significance level was set at *P* < 0.05.

## 3. Results

A total of 122 cases with CA and 122 control subjects without CA were enrolled in this study. Mean age and sex distribution and Fitzpatrick skin phototype of the participants were not significantly different in two groups which have been shown in Table 1.

Mean levels of the total cholesterol, TG, LDL, and HDL were higher in patients with CA compared to control group in which differences were significant for total cholesterol, LDL, and TG (*P* < 0.05) but not for HDL level (*P* = 0.096) (Table 1).

Frequency of dyslipidemia, hypertriglyceridemia, hypercholesterolemia, high LDL levels, and low levels of HDL in the participants has been summarized in Table 2. There was no significant difference in frequency of dyslipidemia between two groups (*P* = 0.27) but there was significant difference in frequency of hypertriglyceridemia, hypercholesterolemia, and high LDL levels between two groups (*P* < 0.05).

Patients with CA were allocated into three subgroups on the basis of CA count: (A) <5 lesions, (B) 5–10 lesions, and (C) >10 lesions; then mean age in these subgroups was compared. In paired comparison, there was a significant difference in mean age between subgroups A and C (Table 3).

Mean levels of total cholesterol, triglyceride, HDL, and LDL were not significantly different in these subgroups (*P* > 0.05).

## 4. Discussion

Senile hemangioma or CA is a vascular tumor which consists of proliferated small vascular channels which have been originated from postcapillary venules in the upper dermis and it has been mentioned that CA should not be considered as a neovascularization [5]. Different etiologies and associations have been proposed for CA which include aging, genetic predisposition, hormonal changes, viral infection, immunosuppression, malignancy, hot climate, diabetes, and some chemical exposures [6–11, 15] but association with dyslipidemia has not been clearly mentioned in prior reports.

We found that hypertriglyceridemia, hypercholesterolemia, and high LDL levels were more frequent in patients with CA compared to control subjects. Mean age in patients with more than 10 CA lesions was significantly higher compared to patients with less than 10 CA lesions.

Increased levels of serum lipids in elderly could be as a triggering factor to release cytokines and chemokines which result in endothelial cell proliferation and CA lesions [11, 13]. Hypercholesterolemia is associated with endothelial cell dysfunction which may be due to toxic products derived from disintegration of lipoproteins [16]. Common pathogenic pathway could be incriminated for CA and dyslipidemia; for example, binding of Insulin-like Growth Factors (IGF) to their receptors results in proliferation of endothelial cells and hyperlipidemia [17]. It has been reported that CA are more and larger in patients with type 2 diabetes mellitus who have more IGF [12, 18] but CA has not been more prevalent in obese patients [19].

Mast cells (MC) may also have a potential role in development of CA and dyslipidemia. MC mediators are triggering factors in neovascularization and the number of mast cells increases in the CA lesions, but these new vascular proliferations may be due to degrading effects of MC mediators on dermal connective tissue [20]. On the other hand, angiogenic agents such as vascular endothelial growth factor (VEGF) have a chemotactic effect on the MC [20–22]. Interestingly it has been shown that MC may have a role in metabolic syndromes and dyslipidemia. It has been reported that chymase and tryptase of the MC could be able to actively degrade HDL. In animal models, it has been shown that mast cell-deficient mice had lower levels of serum triglycerides, total cholesterol, and phospholipids, so presenting a less atherogenic lipoprotein profile [22, 23]. Thus, it seems that MC might be incriminated in development of CA and dyslipidemia simultaneously.

CA has been associated with hypertension and varicosity in old ages [3] and hypertension by itself has been considered as a feature of metabolic syndrome [24] but we had to exclude hypertensive patients in our research because antihypertensive agents could be able to change lipid profile so it should be considered as a confounding factor. Also inflammatory factors decrease lipoprotein lipase activity in autoimmune skin diseases such as lichen planus, psoriasis, or pemphigus vulgaris which may result in dyslipidemia so we excluded these patients from this research [25].

Aging is associated with increasing in prevalence of dyslipidemia and CA, so we tried to have almost equal mean ages in both groups to minimize this confounding factor in our study.

We did not find any association between CA and skin phototype in this research. CA could be seen in any region of the body; thus it seems that sun exposure and skin phototype may not have major roles in development of CA. However it has been proposed that chronic sun exposure could be able to decrease skin tonicity and elasticity, so fragility of the blood vessels would be increased which results in the development of CA, telangiectasias, and senile purpura in elderly [26], but this theory could not justify development of CA on covered body areas and rejects the theory which says CA is a true vascular proliferation [5].

Frequency of hypercholesterolemia, hypertriglyceridemia, and high levels of LDL was lower and frequency of low levels of HDL was higher in our study compared to estimated prevalence of dyslipidemia in Iranian people [14]. Unfortunately dyslipidemia has a higher prevalence among Iranian adults compared to US adults due to higher proportion of carbohydrates in Iranian diet [27].

In summary we think that CA lesions may be a sign of hypercholesterolemia or even a metabolic syndrome, but we had following limitations in our study: relatively small number of subjects was included which may not be fully representative of the general population. Also physical examination of genital and scalp areas was not done for patient's comfort which may result in control selection bias; many older patients were obligatorily excluded due to intake of antihypertensive and lipid lowering agents and other criteria for metabolic syndromes including body mass index were not considered.

## 5. Conclusion

Many different agents have been incriminated in development of CA. Although frequency of dyslipidemia was not significantly different in two groups, hypercholesterolemia and hypertriglyceridemia were more prevalent in patients with CA, so it seems logical to screen lipid profile in subjects with CA to prevent its atherogenic effect in the future.

## Figures and Tables

**Table 1:** Characteristics of participants in the case and control groups.

Variable^*∗*^	Case *N* = 122	Control *N* = 122	*P* value
Age (year)	43.98 ± 9.11	43.48 ± 11.55	0.21^*∗∗*^
Sex			
Male	33 (27%)	44 (36.1%)	0.13^*∗∗∗*^
Female	89 (73%)	78 (63.9%)	
Skin phototype			
2	12 (9.8%)	8 (6.6%)	0.476^*α*^
3	54 (44.3%)	50 (41%)	
4	52 (42.6%)	62 (50.8%)	
5	4 (3.3%)	2 (1.6%)	
Levels of serum lipids (mg/dl)			
Triglyceride	167.37 ± 86.38	144.8 ± 105.40	0.004^*✕*^
Cholesterol	200.39 ± 43.21	175.05 ± 38.15	<0.001^*✕*^
LDL	117.52 ± 29.31	100.64 ± 31.30	<0.001^*✕*^
HDL	50.24 ± 25.28	45.58 ± 13.52	0.096^*✕*^

^*∗*^Variables were described by number (percent) or mean ± standard deviation; ^*∗∗*^Mann-Whitney, ^*∗∗∗*^Chi square, ^*α*^Fisher's exact test, ^*✕*^Kruskal-wallis.

**Table 2:** Lipid profile of participants in case and control groups.

Lipid profile	Case Number (%)	Control Number (%)	Total subjects Number (%)	*P* value^*∗*^
Dyslipidemia				
No	22 (18)	29 (23.8)	51 (20.9)	0.27
Yes	100 (82)	93 (76.2)	193 (79.1)	
Hypertriglyceridemia				
No	61 (50)	82 (67.2)	143 (58.6)	0.006
Yes	61 (50)	40 (32.8)	101 (41.4)	
Hypercholesterolemia				
No	70 (57.4)	91 (74.6)	161 (66)	0.005
Yes	52 (42.6)	31 (25.4)	83 (34)	
High LDL				
No	86 (70.5)	103 (84.4)	189 (77.5)	0.009
Yes	36 (29.5)	19 (15.6)	55 (22.5)	
Low HDL				
No	60 (49.2)	52 (42.6)	112 (45.9)	0.304
Yes	62 (50.8)	70 (57.4)	132 (54.1)	

^*∗*^Chi-square.

**Table 3:** Mean age in subgroups of patients with cherry angioma.

Subgroups of case group	Number of patients	Mean age (year) ± standard deviation	*P* value^*∗*^
A (<5 lesions)	71	41.82 ± 8.38^a^^*∗∗*^	0.002
B (5–10 lesions )	23	44.09 ± 8.26^ab^	
C (>10 lesions)	28	49.36 ± 9.58^b^	

^*∗*^Kruskal–Wallis. ^*∗∗*^Similar characters represent nonsignificant differences of mean age in subgroups.

## Data Availability

The raw data used to support the findings of this study are available from the corresponding author upon request.
